# Cross-border health data sharing between Singapore and Switzerland: controlling for competing regulatory requirements

**DOI:** 10.1093/jlb/lsaf021

**Published:** 2025-10-28

**Authors:** James Scheibner, Hui Yun Chan

**Affiliations:** College of Business, Government and Law, Flinders University, Ring Road, Bedford Park, Adelaide, South Australia 5042, Australia; Centre for Biomedical Ethics, National University of Singapore, 10 Medical Dr, #02-03 MD 11, Singapore 117597

**Keywords:** cross-border data transfer, data protection law, data sharing, GDPR, health data, PDPA

## Abstract

Research in biomedical and health sciences using data-intensive methods increasingly involve multi-party cross-border institutional collaborations. Regulatory complexities governing international data flow remain challenging to navigate, particularly where differing legal standards in relation to data and privacy protections exist in the respective jurisdictions. In this paper, we use the example of a use case from a joint health research program between Singapore and Switzerland to illustrate the possibility of cross-border data flow for these two jurisdictions with no reciprocal adequacy recognition standards. We have therefore compared data privacy and biomedical research ethics laws in both jurisdictions to help determine when cross-border data sharing could occur that are compliant with data privacy laws. Our comparison makes reference to when technical and organizational measures including privacy enhancing technologies are appropriate to support data sharing. This paper has the potential to inform researchers collaborating with international institutions in navigating similar privacy regulatory considerations in their research and in developing their collaborative agreements.

## I. INTRODUCTION

Research on perceptions of data sharing has shown that stakeholders value a range of ethical principles with respect to collection, use, and transfer of health data.^1^ For example, a hospital sharing health data for government funded research would most likely be considered an appropriate flow of information. By contrast, a hospital sharing patient data for privately funded research, or sharing data without consent, is much more likely to violate expectations of contextual integrity.^2^ Applying this contextual integrity framework becomes significantly more complicated when considering cross-border transfers of sensitive health data. On the one hand, biomedical and health sciences research increasingly depends on multi-site, cross-institutional collaborations. The quantity and quality of data generated through these collaborations can lead to significantly improved research outcomes.^3^ On the other hand, some jurisdictions may have regulations governing appropriate flows of data that may not be acceptable in other jurisdictions.^4^ For example, research has shown that differing socio-political environments have the potential to influence the transfer of data between international jurisdictions, particularly on concerns related to data sovereignty and data localization.^5^ Additionally, at a national level, factors such as social license and public trust could shape public attitudes toward sharing their personal data.^6^ For example, a study from Singapore revealed that the social license for sharing precision medicine data supports the sharing with public institutions for health research but not for private commercial use.^7^ Against this backdrop, the legislative intent of data protection frameworks shed further light into the socio-political factors that enable or impede cross-border data flow. For instance, the Personal Data Protection Act of Singapore is intended to balance the right to data protection and businesses’ compliance costs while enhancing Singapore’s competitiveness as a major trading hub.^8^ This multi-pronged aim suggests a pro-market attitude toward data protection to spur economic growth by enabling cross-border data flow while being aligned with broader international legislative frameworks governing data protection and privacy.

The purpose of this paper is to therefore compare research and data privacy norms between two jurisdictions. Such a comparison can help determine when data can be shared across borders between two jurisdictions. A similar approach known as ‘normscreening’ has been used by state governments in Germany to analyze barriers for intra-state data sharing between Saarland and Schleswig-Holstein.^9^ This approach has also been used by McLennan and others to analyze the norms governing the secondary use of COVID-19 data in Bavaria. For the purposes of this paper, we have chosen to focus our analysis on Singapore and Switzerland. Both countries have data privacy and other laws that govern how data can be collected, used, and transferred. However, Singaporean law does not recognize other jurisdictions as providing an equivalent standard of data privacy. Therefore, personal data can only be shared according to certain rules. Conversely, Switzerland only recognizes laws in certain jurisdictions as adequately protecting privacy. The effect of this regulatory inconsistency is that data cannot freely flow between these two jurisdictions without contractual measures in place. Most of standard form contractual measures that have been published by regulatory agencies have been directed at cross-border data sharing for commercial purposes.^10^ By contrast, appropriate data flows for multisite health data research across jurisdictions may be substantially different. Failure to consider this different context may undermine the social license and public trust that is essential for public engagement in data sharing initiatives.^11^

Therefore, our paper is composed of two sections. In the first section, we will examine the differences in how Singaporean and Swiss law treat key concepts in health data privacy. First, we will address how each jurisdiction defines personal data and when data becomes personal data. Second, we will address how each jurisdiction uses different terms such as ‘anonymised’, ‘pseudonymised’, or ‘de-identified’. Third, we will address the grounds upon which the legislation in each jurisdiction permits the cross-border transfer of personal data. In the second section, we will consider how these requirements might inform the flows of health data for research purposes between two jurisdictions.

As a case study, we will use the research conducted as part of the Future Health Technologies program at the Singapore-ETH Centre as an example. Specifically, we will consider what types of data might become personal data under Singaporean or Swiss law, as well as what technical measures exist for anonymizing this data. We will then address how the data flows for a multi-site research project located between the two jurisdictions could operate while remaining compliant with data privacy laws. To this end, we will consider the use of both technical and organizational measures. The goal of this paper will be to inform researchers embarking on multi-site research projects and indicate how they should construct their collaborative agreements.

## II. COMPARING THE TWO DATA PRIVACY AND RESEARCH GOVERNANCE REGIMES

### II.A. Singaporean Law

#### 1. The Regulatory Landscape

The main Singaporean law governing personal data, including sensitive data such as health data, are the *Personal Data Protection Act* (PDPA) and associated regulations. These laws only apply to private sector entities, universities, and hospitals as opposed to government agencies.^12^ In addition, the *Human Biomedical Research Act* (HBRA) governs medical and health sciences research, including research involving individually identifiable health information.^13^ There are also certain types of research that are not regulated under the HBRA. These include national public health research, epidemiological research conducted by the National Registry of Diseases, statistical processing of health information, or clinical trials of health or medicinal products.^14^ The Singaporean Personal Data Protection Commission (PDPC) is the main government agency responsible for enforcing this regulatory framework. Outside of this regulatory framework, there are laws governing the use of personal data by government agencies, including for law enforcement and security purposes. These acts include the *Registration of Criminals (Amendment) Act*, which has recently been amended to allow for law enforcement to collect health and genetic information.^15^

#### 2. The Meaning of Personal Data in Singapore

Under the PDPA, data becomes personal when it can be used to identify an individual, either alone or in concert with other information.^16^ To demonstrate the operation of this provision, the PDPC has previously held that emails or text messages cannot constitute personal information. However, if these messages could be combined with other information to identify individuals (such as email addresses or text messages), they will become personal data. Likewise, residential addresses are not by themselves personal information, as they may be occupied by multiple individuals.^17^ However, if combined with other pieces of identifying information, they may become personal data. As we will discuss in the second section of this paper, this requirement demonstrates how data may become personal data depending on who possesses that data and what they can access.

#### 3. Anonymized Data, Anonymization Techniques, and Assessing Risk of Re-identification

All organizations under the PDPA need to ensure that they maintain the security of personal information.^18^ If an organization no longer possesses the means to re-identify individuals from a dataset, they will possess anonymized data, as opposed to personal data, for the purposes of the PDPA.^19^ Therefore, anonymizing personal data might constitute a strategy for cross-border transfer of health data. What exactly constitutes anonymized data is not defined in either the PDPA or the HBRA (although both acts prohibit re-identifying anonymized data).^20^ The PDPC’s guidelines on anonymization distinguish it from de-identification, two terms that are sometimes problematically used as synonyms in published literature.^21^ The PDPC notes that de-identifying data only involves removing direct identifiers, such as full names and government identifiers.^22^ A dataset that has been de-identified in this manner could still contain personal data if that dataset was combined with other data (including publicly available data). By contrast, the PDPC defines anonymization as a risk management protocol that involves determining whether there is a serious risk that individuals can still be re-identified from the dataset. This risk assessment requires considering both the data itself and the measures or safeguards used to mitigate the risk of individuals being re-identified.^23^

The PDPC does not prescribe the use of specific anonymization techniques. However, the PDPC’s guidelines state that datasets will be considered de-identified when all direct identifiers have been removed. The data controller should also remove all indirect identifiers that could be combined with publicly available information to identify individuals.^24^ The PDPC’s guidelines provide that the status of data as ‘anonymised’ depends as well on the use of additional safeguards. These safeguards can apply to both the recipient of the data as well as members of the organization handling the data. For example, these safeguards could limit the number of individuals who can access, use, or disclose the data, as well as controlling how those individuals can use or disclose the data.^25^

Beyond these requirements, the PDPC does provide a guide on basic anonymization techniques for organizations. Some of the techniques suggested by the PDPC have been used for research related purposes, including *suppression*, *pseudonymization*, *generalization*, *swapping*, *perturbation*, and *aggregation*. A description of each of these techniques is provided in Table 1. *Suppression* and *pseudonymization* are frequently used to remove direct identifiers such as names, identification numbers, and account numbers. Other techniques, such as *generalization*, *swapping*, *perturbation*, and *aggregation*, are more frequently used on indirect identifiers, such as age or location. The PDPC’s guidelines also suggest that different techniques should be applied to different use cases. These use cases include internal data sharing of both de-identified and anonymized data internally, external data sharing, long-term data retention, and generating *synthetic data*.^26^

**Table 1:** Common de-identification and anonymization techniques and terms

Suppression	Removing certain records from a dataset that may be re-identifiable based on a given set of attributes before release^27^
Pseudonymization	Replacing direct identifiers such as names, identification numbers, and account numbers with pseudonyms^28^
Swapping	Randomly swapping values (usually randomly) between different records^29^
Generalization	Replacing specific values with a general value in a certain range to reduce the risk of an individual being re-identified from that value^30^
Perturbation	Adding ‘noise’ to the dataset by modifying specific values to reduce the risk of individuals being re-identified from indirect identifiers or unusual values^31^
Aggregation	Providing summarized values about a particular population or group of records^32^
Synthetic data	Data that allows the same inferences to be generated as they would be from real data but do not contain any personal data.^33^
*k*-Anonymity	A property possessed by a dataset that has been de-identified. A dataset will have reached a certain *k* value if there are at least *k* − 1 other records in the dataset that share attributes and are therefore indistinguishable from one another^34^
Cryptographic hash	An algorithmic technique for turning a string of characters, or an input, into an encrypted value, or a digest^35^
Differential privacy	A mathematical framework for releasing aggregate data while limiting what can be inferred about any one individual in a dataset^36^
Trusted execution environment	Trusted execution environments allow data to be processed in an encrypted space on a computer that cannot be accessed without an encryption key^37^
Homomorphic encryption	An advanced encryption technique that allows a third party to perform operations on a dataset and receive the aggregate results of those operations without necessarily receiving the underlying information^38^

With respect to the risk of identification from certain datasets, the PDPC’s guidelines note that some types of data may contain more information than others. If a dataset contains highly sensitive information, the organization holding that data should reduce the granularity of that dataset to reduce the risk of harm. This focus on sensitivity is reflected in other areas of the PDPA. The *Personal Data Protection (Notification of Data Breaches) Regulations 2021* lists categories of sensitive health information, which, if breached, must be reported to the PDPC. These include information on sexually transmitted diseases, certain mental health conditions, substance abuse and addiction, sperm or egg donation, contraceptive procedures, or organ donation. When handling these and other highly sensitive personal data, the PDPC’s guidance recommends reducing the granularity of data to reduce the risk of harm from re-identification. In addition, the PDPC notes that *k-anonymity* can be used to assess a dataset that contains microdata, or records belonging to individuals, prior to release. The PDPC provides that if data is anonymized to a minimum *k*-anonymity value of 5, it can be shared with external organizations provided that the internal safeguards are in place. Data that is to be shared internally can be anonymized to a minimum *k*-anonymity value of 3.^39^ The PDPC’s guidelines note that these values are generated from equivalent Australian guidance on data quality and de-identification.^40^

#### 4. Using Personal Data for Research Purposes

By default, under the PDPA personal data can only be collected, used, or disclosed with the consent of the individual to whom it relates.^41^ When collecting this data, an organization must inform the individual of the reasons why they are collecting, using, or disclosing their personal information. Alternatively, the PDPC recommends that research organizations should use non-personal or anonymized data where possible to reduce the risk of data breaches.^42^ However, the Second Schedule of the PDPA also provides four criteria for when personal data can be used or disclosed without consent for research.^43^ First, the research purpose cannot reasonably be accomplished unless the personal data is identifiable. Second, there must be a clear public benefit to using the personal data for the research purpose. Third, the results of the research may not be used to make decisions that affect individuals in that dataset. Fourth, the results must be published in a form that does not identify an individual.^44^ The same four requirements apply to disclosing information for research purposes, except that it must also be impractical to seek consent for the research purpose.^45^ The PDPC has indicated what factors would determine whether it would be impractical to seek consent. These include where the organization lacks the current contact details of research participants, where it would be impractical to seek consent given research funding, or whether there are exceptional circumstances. These exceptional circumstances could include situations where seeking consent would undermine the ability of the research team to recruit participants.^46^

Any research project involving health information would need to comply with the laws around consent under the HBRA. Although appropriate consent must be taken from research participants,^47^ an institutional review board can waive the requirement to seek consent for research with health information.^48^ Similarly to the PDPA Schedule 2, the institutional review board must be satisfied the research requires identifiable health information and it would take disproportionate effort to contact participants.^49^ In addition, the institutional review board must be satisfied if there is only minimal risk to participants from the research and no adverse impact on rights or welfare.^50^ The research must also contribute to the greater public good.^51^

In addition, the PDPC has provided practical guidance in two circumstances concerning lawful grounds for processing personal data in research. The first was a request by several organizations and a public agency in a data collaboration agreement attempting to ‘address social well-being issues through the use of anonymised or synthetic datasets’.^52^ The data collaboration agreement involved a team from the public agency identifying a population of interest and defining a *salt*, or an additional input of random data, to generate a *cryptographic hash*. This hash was then provided to each of the organizations to generate a pseudonymized dataset using the data that each organization held about that population. Another team from the public agency then was responsible for fusing each of these datasets together into either an anonymized or *synthetic* dataset.^53^ The collaboration approached the PDPC on whether the organizations bound by the PDPA were required to seek consent to disclose the pseudonymized data and to merge the data into an anonymized form. The PDPC concluded that the organizations did not need to seek consent to either disclose or merge these datasets. However, the PDPC noted that whether the data remained anonymized would depend on where the data was shared. The second was a request by a medical research institution to clarify when it would be impractical to seek consent for the purposes of Parts 2 and 3. The PDPC held that factors like mere inconvenience, as well as additional costs or time delays, are insufficient to demonstrate impracticality.^54^ If the research became onerously expensive because of contacting participants, this cost may meet the threshold of impracticality. The PDPC also held the perspectives of institutional review boards or ethics committees would be pertinent in determining what a reasonable researcher would consider impractical. Nevertheless, the PDPC held that an ethics committee could not waive the requirement for researchers to comply with the PDPA. Therefore, even if an institutional review board awards a waiver of consent, a research institution will still need to comply with the legal rules on transfer.

#### 5. Cross-Border Transfer of Personal Data

Under the PDPA, personal data can only be transferred from Singapore to other jurisdictions in accordance with the regulations.^55^ This requirement only applies to personal data and not anonymized data. However, the organization based in Singapore should determine whether the data meets the test for anonymized data in the receiving jurisdiction. If it does not, it may be treated as personal data in the receiving jurisdiction.^56^ Therefore, it is possible that even if data is anonymized to a minimum *k*-anonymity value, it may still fall within the remit of the PDPA if other privacy enhancing technologies are not used. The *Personal Data Protection Regulations* (PDPR) provide that a transferring organization must ascertain whether the recipient jurisdiction offers equivalent privacy protection to Singapore. The transferring organization must then determine whether the recipient organization is bound by legally enforceable obligations.^57^ This requirement can be satisfied through a number of legal avenues. First, data can be transferred if the individual to whom the personal information belongs to consents or is deemed to have consented to the transfer.^58^ Second, personal data can be transferred when it is clearly in the interests of an individual and where consent cannot be obtained in a timely way.^59^ It is also possible to transfer an individual’s personal information where there is an emergency that threatens the life, health, or safety of that individual.^60^ Personal data can also be transferred if it is data in transit or is publicly available in Singapore.^61^ ‘Data in transit’ refers to personal data transferred through Singapore during onward transportation to a country or territory outside of Singapore without the data being accessed or used in Singapore.^62^

These requirements raise the question of when personal data can be transferred outside of Singapore to a third-party jurisdiction. Several recent decisions from the PDPC are determinative on these requirements. In *Spize Concepts Pte Ltd*, the PDPC held that the transferring organization could satisfy the PDPR by ensuring the recipient jurisdiction offered equivalent legal protection. The transferring organization could also satisfy the PDPR by providing the recipient organization with a binding contract imposing Singaporean data security standards.^63^ In the alternative, one of the exceptions provided for in PDPR could be used to justify a transfer. However, commercial choice or convenience will not be sufficient to justify a transfer.^64^ Furthermore, the terms of the agreement must be legally binding; otherwise, the transfer requirement will not be satisfied. In *Belden Singapore Private Limited*, the Singaporean subsidiary of the Belden group had not ratified a deed imposing equivalent data security requirements across all Belden group subsidiaries.^65^ Because Belden Singapore then subsequently signed the agreement, it only received a warning as opposed to penalties.^66^ The requirements for binding regulatory guidelines apply to transfer to all jurisdictions, including to the European Union. In *Toll Logistics Asia Limited and others*, Toll Logistics contracted with a software vendor in Ireland for human resource management, which hosted serves in the European Economic Area. The PDPC held that Toll needed to ensure that the recipient offered equivalent protection to the PDPA prior to that data being transferred outside Singapore.^67^ Therefore, the transfer of any kind of sensitive personal data from Singapore would presumably need to occur in compliance with a data transfer agreement. This data transfer agreement would be required even where the recipient jurisdiction offers an equivalent or higher standard of data protection.^68^ An agreement would not necessarily be required if the participants have agreed to the transfer and have been provided with a reasonable summary of how their personal data will be protected.^69^ However, this approach would require seeking explicit consent from every participant for the transfer. This approach may not be appropriate for large longitudinal datasets.^70^

In addition to these laws, there has been practical guidance published on the meaning of ‘data-in-transit’ by the PDPC with respect to the use of privacy enhancing technology. This practical guidance concerned Zuellig Pharma, which had created a *trusted execution environment* into which third parties could transfer data.^71^ Third-party organizations could then use a web application to hash records in the dataset containing personal or sensitive data, and then access this data themselves. The PDPC held that, provided the hashing was compliant with the PDPC’s guidance on anonymization, transferring data using this method would not constitute a transfer of personal data. Therefore, consent would not need to be obtained from individuals to whom the data relates to.

### II.B. Swiss Law

#### 1. The Regulatory Landscape

Much like Singapore, Switzerland’s data privacy regime with respect to personal health data is provided for by several overlapping pieces of legislation. The Swiss *Datenschutzgesetz*, or Federal Act on Data Protection, governs the processing of personal data in Switzerland, including sensitive personal data such as health data.^72^ The FADP was updated in 2023 to ensure greater regulatory convergence with the European Union’s General Data Protection Regulation (GDPR).^73^ In addition, the Swiss *Humanforschungsgesetz* or Federal Human Research Act (HRA) governs health research involving human data or biological material.^74^

#### 2. The Meaning of Personal Data and Sensitive Data

Under the FADP, personal data includes all information relating to an identified or identifiable natural person.^75^ This definition can be considered roughly equivalent to the definition that exists under Article 4 of the GDPR.^76^ The Article 29 Working Party, the former regulatory authority governing EU law, notes that personal data will be considered related to a person if it satisfies one of three criteria. These three criteria are if the information is about a person, is used to treat a person a certain way, or has an impact on the rights of that person. This definition is broad and would include information such as a person’s professional habits or practices, their car service record, or a call log for a telephone, provided they can be linked to that person.^77^ The FADP also applies to sensitive personal data, including data on a person’s religion, their ideological or political affiliation, their health or ethnic origin, any biometric data that identifies that person, or any data concerning criminal proceedings. The HRA provides that ‘health-related personal data’ means any information concerning the health or disease of a specific person, including their genetic data.^78^

#### 3. Anonymization Techniques, Pseudonymization, and Risk Assessment

The term ‘anonymised’ is not defined in the FADP, but several sections reference this term. For example, the FADP requires data controllers and processors to destroy or anonymize data as soon as it has been processed.^79^ Accordingly, under the FADP anonymized data should be considered equivalent to non-personal data. With respect to the previous FADP, the Federal Data Protection and Information Commissioner (FDPIC) has issued guidelines on when data will be anonymized. These guidelines state that data will only be anonymized when ‘all options of recreating the original data are eliminated completely’.^80^ Furthermore, Martani and others observe that in recent cases before the Swiss Federal Supreme Court, the question of whether data is still identifiable once anonymization has been applied is contextual. Specifically, the Supreme Court held that if an individual has the means to access additional information to combine two datasets together, that data will no longer be anonymized.^81^

However, the HRA defines anonymized health-related data as health-related data that, without significant effort, cannot be traced to a person.^82^ This definition implies that health-related data that may be difficult but not impossible to re-identify could still be considered anonymized. This approach to anonymization is closer to the relative approach adopted under the GDPR and some European Union data protection authorities such as the Irish Data Protection Commission.^83^ The guidelines on the FADP and the HRA published by the FDPIC also note that if the data controller or processor cannot conduct their research without using anonymized data, they should use pseudonymized or coded data.^84^ However, this data should still be treated as personal data. This means that consent must be sought from participants for it to be used for research purposes, subject to the requirements discussed in the next section. Ormond and others note that this regulatory uncertainty over the meaning of personal, coded, and anonymized data is seen as a barrier to data sharing by Swiss researchers. In addition, Ormond and others observe that differences in how pseudonymized or anonymized data is classified in other jurisdictions (namely, the United Kingdom and the USA) is perceived as a barrier for cross-border data sharing.^85^ Likewise, in the second section of this paper we will address how inconsistencies in the Singaporean and Swiss definitions of anonymized and pseudonymized data can represent a challenge for data sharing.

#### 4. Using Personal Data for Research

As with Singapore, by default Swiss data controllers and processors (including researchers) can only use and disclose personal data if it is collected lawfully. The controller or processor must inform the data subject who is collecting the data, as well as the purpose for which it is being collected. If the data is being transferred overseas, then the controller or processor should also provide the data subject with the name of the state or the entity processing that data.^86^ However, the FADP does create exceptions to this requirement for controllers in the private sector and Federal agencies. Private sector controllers carrying out processing of personal information must ensure that the personal data is anonymized or at least pseudonymized if anonymizing the data would take disproportionate effort. The controller conducting the research also must ensure that results are published in such a manner that data subjects are not identifiable. Likewise, sensitive personal data can only be disclosed to third parties in a way that they are not identifiable.^87^ Federal agencies carrying out data processing must also ensure the data is rendered anonymous as soon as possible.^88^ The FDPIC notes that these rules apply to research that does not involve health-related personal data. Research involving human beings may only be carried out with the consent of research participants.^89^

The HRA also creates specific rules for ‘further’ research involving health-related personal data. Article 32 provides that informed consent for further research purposes with genetic or biological data is only required if the data will be used in an uncoded or coded form for research purposes. If anonymized, the data can be used if the person is informed in advance and has not dissented.^90^ Article 33 provides that informed consent further research with non-genetic health-related data is only required if it will be used in an identifiable form.^91^ Otherwise, the data can be used in a coded form if the person concerned has not dissented when informed in advance. ‘Further’ research in this context can be considered equivalent to secondary uses of personal information with opt-out or general consent.^92^ The HRA also provides an exception where it is not possible to fulfill the requirements of Article 32 or 33. In these circumstances, further use may be made of health-related data if it is impossible or disproportionately difficult to obtain consent or obtaining consent would place undue burden on the person collecting the data.^93^ In addition, there must be no documented refusal from the person and the public interest in carrying out the research must outweigh the interests of the individual to whom the data belongs.^94^ Essentially, Article 34 permits a waiver of consent in a similar fashion to the *Human Biomedical Research* Act in Singapore.^95^ Nevertheless, how consent under Articles 32 or 33 or a waiver under Article 34 are implemented in practice depends on the preferences of ethics committees authorizing research.^96^ In addition, the impact of international collaboration and cross-border transfers of data constitutes a further complicating factor.

#### 5. Cross-Border Transfers of Personal and Health Data

Comparably to Singaporean data privacy law, the FADP prohibits the disclosure of personal data abroad to jurisdictions unless the recipient jurisdiction offers adequate data protection. Unlike the PDPA, the FADP defines this disclosure as including transmitting or making personal data available.^97^ This protection can be guaranteed by an international treaty, data protection provisions of a contract, specific safeguards, standard data protection clauses, or binding corporate rules.^98^ However, any provisions, safeguards, clauses, and corporate rules must be approved by the FDPIC. Furthermore, the Federal Data Protection Ordinance (FDPO) published with the FADP provides a list of countries that offer adequate protection. Although Singapore is not listed as one of these countries, the FDPIC notes that it has not assessed the data privacy laws of each jurisdiction. In the alternative, a Swiss organization can derogate from these requirements and transfer personal data if the data subject has explicitly consented to the disclosure abroad.^99^

The FDPIC has also written guidance on what types of data (including personal and non-personal data) can be transferred to jurisdictions, which do not offer equivalent privacy to Switzerland. However, these guidelines apply to the previous version of the FADP.^100^ These guidelines impose requirements on the controller sending the data to the other jurisdiction, otherwise known as the data exporter. First, the data exporter must maintain records about the data being transferred. These records include details about whether the data includes personal data, whether those persons are identifiable, the purpose for transfer, and the categories of personal data being transferred. These records should also include details about whether any third parties responsible for data processing are subject to laws from other jurisdictions. For example, the FDPIC guidelines explicitly refer to the US and cloud computing providers, which may be subject to their own requirements regarding anti-terrorism laws.^101^ Second, the data exporter must check whether fundamental Swiss rights are guaranteed in the country with respect to official access to personal information. These four include clear precise and accessible rules, proportional principles of access, effective legal regimes, and guarantee of legal recourse.^102^ If these guarantees are not available under the law of the target jurisdiction, the data exporter must provide additional measures. These can include both contractual, organizational, and technical measures.^103^ Because a data recipient might not be able to guarantee that they will be forced to comply the law, the first two requirements are unlikely to be sufficient. However, as discussed in the second section of this paper, technical measures may be more appropriate.

### II.C. Critical Analysis of each Regime

The preceding analysis demonstrates that there are two significant differences between the Singaporean and Swiss data privacy regimes. First, the definition of ‘personal data’ is roughly equivalent under both regimes. However, data that may be classified as anonymized under Singaporean law may not be treated as such under Swiss law. The PDPC’s guidelines reference *k*-anonymity as a measure of anonymization. However, *k-anonymity* only provides a theoretical guarantee of privacy. That is, individuals whose records are included in a microdata dataset could still be re-identified despite that dataset meeting a certain *k*-anonymity value. This re-identification could occur through a process known as an inference attack, where a recipient of the dataset possesses other knowledge about individuals in the dataset.^104^ If each record has many attributes, these attributes could easily become what Aggarwal terms ‘quasi-identifiers’, which could then be used to isolate individual records.^105^ The ability to conduct inference attacks may remain even where techniques such as perturbation and generalization are used to reduce the granularity of data. Furthermore, the risk of re-identification via inference increases significantly with respect to longitudinal records, such as the number of times a patient has visited a hospital or other location.^106^ Therefore, there is no guarantee that a dataset with a particular *k-anonymity* value can be anonymized while retaining its usefulness as a dataset. Since these shortcomings of *k-anonymity* have become apparent, other measures of anonymity have been proposed, such as *t-closeness*, *l-diversity*, and *differential privacy*. However, of these measures only differential privacy offers a mathematical rather than a theoretical guarantee of privacy.^107^ We will return to consider the use of different technical techniques for de-identification or anonymization of health data with respect to Swiss data privacy law.

Second, the rules governing data transfer between Singapore and Switzerland are divergent. Specifically, personal data can only be transferred from Singapore either with the consent of the individual affected or if anonymized. Furthermore, any transfer of identifiable personal data would be subject to a contract or an agreement governing transfer, unless explicit consent was sought from the data subject. This requirement would include to a jurisdiction such as Switzerland, which offers a comparable or higher standard of data protection. These consent requirements might be sustainable for projects where it is possible to obtain consent prior to carrying out the research. These projects could include surveys, qualitative research, and randomized control trials. However, this approach would be unsustainable for retrospective quantitative research involving large sets of health data where it would be impractical to obtain consent, such as longitudinal research projects. Conversely, under the FDPO, personal data cannot be disclosed from Switzerland to Singapore without the existence of standard contractual clauses approved by the FDPIC or explicit consent. Alternatively, if non-approved contractual clauses are used to justify a transfer, these transfers must be notified to the FDPIC. It is possible that a future Swiss court or the FDPIC may treat Singapore as offering adequate data privacy laws. However, it should be noted that the PDPA in Singapore only applies to private organizations, universities, hospitals, and not government agencies.^108^ Therefore, it is possible that the PDPA might not meet the four guarantees of fundamental rights specified in the FDPIC’s guidance documents.

These requirements, if not considered appropriately, can create a significant administrative burden for already stretched research consortia and research institutions. This would involve reviewing existing protocols and timeline for data transfer (eg any security requirements or restrictions) and administrative support that are available to facilitate the data transfer process. For example, research ethics committees perform a range of administrative tasks in assessing the benefits and risks of research, including potential data sharing, and which would involve a broader network of administrative entities such as data protection and biomedical research offices. Commercial entities involved in data transfer similarly face compliance and business costs in navigating data transfer laws. For example, the mechanisms in the new Chinese PRC Personal Information Protection Law for transferring personal data abroad has raised concerns regarding costs.^109^ A study from Vietnam similarly noted administrative delays associated with approval processes and costs related to impact assessment and compliance for cross-border data transfer.^110^ Therefore, the next section will consider how scientific research on personal data can be performed between researchers based in Singapore and Switzerland. Although an in-depth discussion of technical measures is beyond the scope of this paper, the next section will consider what technical guarantees exist. It will also consider how these can be combined with legal strategies to facilitate the exchange of different types of data between the two jurisdictions.

## III. STRATEGIES FOR HEALTH DATA SHARING BETWEEN JURISDICTIONS WITH INCOMPATIBLE PRIVACY LAWS

### III.A. Preliminary Requirements

The first step for any transfer of personal data or anonymized data from Singapore to Switzerland would be to put in place an agreement between the provider and recipient of the data. This agreement would be necessary to ensure that the rights of individuals whose data has been transferred are protected in the recipient jurisdiction. This agreement would also ensure that an appropriate mechanism is in place to respond to any data breaches, as well as which regulatory agencies would be responsible. It should be noted that data can still be lawfully transferred from Singapore to Switzerland or from Switzerland to Singapore if consent is obtained.^111^ However, this may not be an appropriate strategy for all research projects.

### III.B. Sharing Data from Singapore to Switzerland

#### 1. Non-Personal Data

If data does not relate to an individual, it can be transferred without consent. Although the standard of anonymization is higher under Swiss than Singaporean law, data such as aggregate survey data that does not contain identifying information can be shared.^112^ Nevertheless, the challenge with sharing aggregate information is that there is a possibility of residual re-identifiability.^113^ Data privacy laws do not provide guidance on how aggregated data must be before it ceases to become personal data.^114^ If there are individuals with a rare disease type that respond to a survey, this may increase the risk of re-identifiability.^115^ In these cases, other mechanisms may be required to preserve privacy for data transfer. For example, differential privacy can be used as a mechanism to add noise to a dataset so that the risk of data disclosure about an individual can be mathematically nullified. However, there are important compromises to be considered when using differential privacy. On the one hand, adding noise to a dataset decreases the statistical utility of a dataset. On the other hand, a non-expert user may not be able to add sufficient noise to eliminate the risk of incidental disclosure.^116^ Therefore, differential privacy may need to be used in concert with other advanced privacy enhancing technologies.

#### 2. Anonymized and Pseudonymized Data

As discussed previously with respect to *k*-anonymity as a measure of identifiability, this risk of re-identifiability rises with multiple data releases.^117^ Therefore, whether the aggregate data may contain identifiable data depends on what other identifiable information has been released alongside this data. Likewise, there is a risk of re-identification with other forms of data, including individual level microdata as well as images and video data. One strategy that can be used to control what data is released to collaborators is with privacy-enhancing technologies. Both software-based solutions such as *homomorphic encryption* and hardware-based solutions such as *trusted execution environments* may enable the transfer of medical data across borders and between organizations.^118^ Crucially, if the recipient organization does not possess the encryption key, they will not be able to re-identify individuals in the dataset, and from their perspective the data will remain anonymized.

The GDPR has similar adequacy provisions to the FADP that can represent a challenge for researchers located in ‘third party’ jurisdictions.^119^ However, Juliussen and others argue that in some cases, privacy-enhancing technologies such as *homomorphic encryption* and *trusted execution environments* could be used to ensure compliance with the transfer provisions of the GDPR.^120^ Likewise, the previously discussed precedent from the PDPC confirms that personal data cannot be shared to third-party jurisdictions without a contractual agreement to govern that data. Therefore, privacy-enhancing technologies could provide a mechanism to reinforce these contractual provisions. These technologies could also ensure that a research organization in Singapore is compliant with its data protection obligations under the PDPA. Finally, under the PDPA a waiver of consent may be sought in situations where it is not possible to obtain consent from participants. The PDPC’s guidance has indicated that a waiver would not remove an organization’s obligations under the transfer rules. However, privacy-enhancing technologies could provide these security guarantees and reduce the risk that personal information would be disclosed.

#### 3. Personal Data

By contrast, in other, small-scale studies, consent to transfer can be obtained from participants in advance, such as for qualitative studies. In these circumstances, given the robust consent provisions in both Switzerland and Singapore, the most straightforward pathway for transfer is consent. That is, prior to collecting any personal data from participants, the research team should inform the participants of where their data will be sent and processed. The consent form should also highlight who the research participants can contact to discuss their study. Furthermore, a consent form does not remove the requirement for research teams to keep data secure. Therefore, some of the privacy-enhancing technologies described in the previous section could still be used to keep data secure.

### III.C. Sharing Data from Switzerland to Singapore

As discussed previously, the requirements for transfer of data are significantly more stringent under the FADP than the PDPA. In particular, consent from research participants is not sufficient grounds to transfer personal data from Switzerland to another jurisdiction. Therefore, it may not be appropriate to transfer personal data from Switzerland to Singapore for further data processing. There could be an exception where some of the privacy-enhancing technologies described previously are used to limit transfers to aggregate computations. In addition, data visiting, where data is analyzed in a provider’s computing environment, has been proposed as a solution to the challenges with cross-border sharing of genomic data.^121^ However, the use of data visiting would be subject to the same technical considerations as discussed above. Furthermore, it is not clear whether data visiting implemented through temporary viewing of data via a *trusted execution environment* would constitute disclosure under data privacy laws such as the FADP.^122^ To prevent any legal concerns, it may be necessary to combine privacy-enhancing technologies to limit access to personal data in such an environment.

### III.D. Broader Application and Implications

The proposed strategies involving establishing data transfer agreements between participating institutions and obtaining consent (in the case of small-scale studies) are part of routine research collaborations tasks and the administrative impact is likely to be minimal. Introducing privacy-enhancing technology could potentially incur additional costs in both software installation/maintenance/upgrade and manpower (expertise) however the long-term returns to the organizations in terms of meeting regular compliance auditing and reaping reputational rewards, which could potentially translate to public trust could incentivize them to implement this aspect. While the use case illustrated above is discussed in the context of Future Health Technology program at SEC, the strategy is capable of being broadened to other research projects within the program, for example, in research projects involving the sharing or transferring of health and lifestyle data generated from mobile apps and information generated by stroke rehabilitation exoskeleton models. Both involve the use and analyses of personal data to assist in supporting health treatments and interventions and further research, and beyond Singapore and Switzerland. Beyond the research program, the strategy is likely to be relevant to research programs involving sharing of data between different cantons in Switzerland with various governance levels of data protection. For example, in a study exploring mobile health adoption in the Swiss health system, one of the reported barriers for adoption was data privacy and safety (eg permissions to access, permissibility of data transfer to third parties).^123^ Another potential area of application is the sharing of genomic data for research as part of large-scale international collaborations between multiple countries. For example, the Global Alliance for Genomics and Health has referred to the importance of establishing cloud privacy and security policy framework in facilitating secure, privacy-preserving federated analysis.^124^ Other relevant areas of research that could benefit from the proposed strategy include international collaborations for research in rare diseases, treatment evaluation for drug developments, personalized medicine, or medical imaging.

## IV. CONCLUSION

International cross-border research collaborations create opportunities for scientific developments that leverage on the diversity of health datasets from different jurisdictions. It also presents legal and technical challenges especially in meeting distinct regulatory requirements in the respective jurisdictions related to data protection and privacy expectations. In this paper, we have offered some guidance for researchers who are considering multi-site research projects that could be used as strategies in constructing their collaborative agreements to address concerns that arise where transfer of health data is proposed. The differences in the jurisdictional treatment of personal data between Singapore and Switzerland demonstrate concrete challenges of transferring health data in the absence of reciprocal adequacy recognition standards and where interpretations of data anonymity and privacy differ. Obtaining consent from research participants prior to data transfer remains a key requirement in both jurisdictions. Our proposed strategy identified two key measures: the use of contractual agreements in addition to ensuring that consent requirements are met and the application of privacy-enhancing technology. Contractual agreements provide a clear indication of how collaborators handle personal and health data for research in accordance with data protection rules in each jurisdiction. The use of privacy-enhancing technology in processing data is expected to mitigate concerns that might arise in relation to residual identifiability of personal data, while demonstrating that appropriate measures are adopted in meeting minimum technical thresholds of anonymization. Both measures can be implemented at the institutional level, with appropriate documentation and administrative support to aid researchers whilst minimizing administrative burden.

## Footnotes

1 Frederic Gerdon et al., *Individual Acceptance of Using Health Data for Private and Public Benefit: Changes During the COVID-19 Pandemic*, Harvard Data Sci Rev., 4–5 (2020), https://hdsr.mitpress.mit.edu/pub/3uc0p5dq/release/5; Henning Silber et al., *A Preregistered Vignette Experiment on Determinants of Health Data Sharing Behavior: Willingness to Donate Sensor Data, Medical Records, and Biomarkers*, 41 Politics Life Sci., 161, 162–6 (2022).

2 Elizabeth Hutchings et al., *A Systematic Literature Review of Health Consumer Attitudes towards Secondary Use and Sharing of Health Administrative and Clinical Trial Data: A Focus on Privacy, Trust, and Transparency*, 9 Syst. Rev. 235, 37–8 (2020).

3 James Scheibner et al., *Data Protection and Ethics Requirements for Multisite Research with Health Data: A Comparative Examination of Legislative Governance Frameworks and the Role of Data Protection Technologies*, 7 J Law Biosci, 1–2 (2020), https://academic.oup.com/jlb/article/7/1/lsaa010/5825716.

4 Bonnie Kaplan, *How Should Health Data Be Used?: Privacy, Secondary Use, and Big Data Sales*, 25 Camb. Q. Healthc. Ethics 312, 325 (2016).

5 Luca Belli, Water B. Gaspar & Shilpa Singh Jaswant, *Data Sovereignty and Data Transfers as Fundamental Elements of Digital Transformation: Lessons from the BRICS Countries*, 54 Comput. Law Secur. Rev. 106017, 9 (2024); Nigel Cory, Cross-Border Data Flows: Where Are the Barriers, and What Do They Cost? 2 (2017), https://itif.org/publications/2017/05/01/cross-border-data-flows-where-are-barriers-and-what-do-they-cost/.

6 Sam H. A. Muller et al., *The Social Licence for Data-Intensive Health Research: Towards Co-Creation, Public Value and Trust*, 22 BMC Med. Ethics 110, 2 (2021); Tamra Lysaght et al., *An Ethical Code for Collecting, Using and Transferring Sensitive Health Data: Outcomes of a Modified Policy Delphi Process in Singapore*, 24 BMC Med. Ethics 78, 2–3 (2023); Tamra Lysaght et al., *“ Who Is Watching the Watchdog?”: Ethical Perspectives of Sharing Health-Related Data for Precision Medicine in Singapore*, 21 BMC Med. Ethics 118, 2–3 (2020).

7 Tamra Lysaght et al., *Trust and Trade-Offs in Sharing Data for Precision Medicine: A National Survey of Singapore*, 11 J Pers Med 921, 7 (2021).

8 Warren B. Chik & Keep Ying Joey Pang, *The Meaning and Scope of Personal Data under the Singapore Personal Data Protection Act*, 26 SAcLJ 354, 356, 365 (2014).

9 Stuart McLennan et al., *Practices and Attitudes of Bavarian Stakeholders Regarding the Secondary Use of Health Data for Research Purposes During the COVID-19 Pandemic: Qualitative Interview Study*, 24 J. Med. Internet Res. e38754, 8 (2022).

10 Warren B. Chik, *The Singapore Personal Data Protection Act and an Assessment of Future Trends in Data Privacy Reform*, 29 Comput. Law Secur. Rev. 554, 565 (2013); Benjamin Wong YongQuan, *Data Privacy Law in Singapore: The Personal Data Protection Act 2012*, 7 International Data Privacy Law 287, 300 (2017).

11 James Scheibner, Marcello Ienca & Effy Vayena, *Whose Health Record? A Comparison of Patient Rights under National Electronic Health Record (NEHR) Regulations in Europe and Asia-Pacific Jurisdictions*, 2021 Sing. J. Legal Stud. 56, 58 (2021).

12 Personal Data Protection Act *2012*, section 3.

13 Human Biomedical Research Act *2015*, section 3(2)(d).

14
*Id.* at Second Schedule, Clauses 1 to 7.

15 Hui Yun Chan et al., *Accessing Third-Party Research Databases for Criminal Investigations: Enhancing Legal Protections and Safeguarding Public Interests*, 24 Med. Law Int. 97, 102 (2024).

16
*Supra* note 12 at 2.

17
Advisory Guidelines on Key Concepts in the PDPA (2022), https://www.pdpc.gov.sg/guidelines-and-consultation/2020/03/advisory-guidelines-on-key-concepts-in-the-personal-data-protection-act.

18
*Supra* note 12 at section 4(3).

19
Personal Data Protection Commission, Advisory Guidelines on the PDPA for Selected Topics (2024), https://www.pdpc.gov.sg/-/media/files/pdpc/pdf-files/advisory-guidelines/ag-on-selected-topics/advisory-guidelines-on-the-pdpa-for-selected-topics- (revised-may-2024).pdf.

20
*Supra* note 12 at section 48F; *supra* note 13 at section 28.

21 Amicia Phillips, Pascal Borry & Mahsa Shabani, *Research Ethics Review for the Use of Anonymized Samples and Data: A Systematic Review of Normative Documents*, 24 Account. Res. 483, 488 (2017).

22
Personal Data Protection Commission, *supra* note 19 at 10–11.

23
*Id.* at 11–12.

24
*Id.* at 11.

25
*Id.*

26
Guide to Basic Anonymisation (2022), https://www.pdpc.gov.sg/-/media/Files/PDPC/PDF-Files/Advisory-Guidelines/Guide-to-Basic-Anonymisation-31-March-2022.pdf.

27 Duncan Smith, *Re-Identification in the Absence of Common Variables for Matching*, 88 International Statistical Review 354, 358 (2020).

28
Guide to Basic Anonymisation, *supra* note 26.

29 William E. Winkler, *Masking and Re-Identification Methods for Public-Use Microdata: Overview and Research Problems*, *in*  Privacy in Statistical Databases 231, 233 (Josep Domingo-Ferrer & Vicenç Torra eds., 2004).

30
Simson L. Garfinkel, De-Identification of Personal Information 20 (2015), https://www.nist.gov/publications/de-identification-personal-information.

31
Guide to Basic Anonymisation, *supra* note 26.

32 Bradley Malin, David Karp & Richard H. Scheuermann, *Technical and Policy Approaches to Balancing Patient Privacy and Data Sharing in Clinical and Translational Research*, 58 J. Investig. Med. 11, 14–15 (2010).

33 John M. Abowd & Julia Lane, *New Approaches to Confidentiality Protection: Synthetic Data, Remote Access and Research Data Centers*, *in*  Privacy in Statistical Databases 282, 283–4 (Josep Domingo-Ferrer & Vicenç Torra eds., 2004).

34 Pierangela Samarati, *Protecting Respondents Identities in Microdata Release*, 13 IEEE Trans. Knowl. Data Eng. 1010, 1012 (2001); Latanya Sweeney, *K-Anonymity: A Model for Protecting Privacy*, 10 Int. J. Unc. Fuzz. Knowl. Based Syst. 557, 564–5 (2002).

35 Christof Paar & Jan Pelzl, *Hash Functions*, *in*  Understanding Cryptography: A Textbook for Students and Practitioners 293, 296 (Christof Paar & Jan Pelzl eds., 2010), https://doi.org/10.1007/978-3-642-04101-3_11.

36 Cynthia Dwork et al., *Calibrating Noise to Sensitivity in Private Data Analysis*, *in*  Theory of Cryptography 265, 265 (Shai Halevi & Tal Rabin eds., 2006).

37 Mohamed Sabt, Mohammed Achemlal & Abdelmadjid Bouabdallah, *Trusted Execution Environment: What It Is, and What It Is Not*, 1 *in* 2015 IEEE Trustcom/BigDataSE/ISPA 57, 57–8 (2015), https://ieeexplore.ieee.org/document/7345265.

38 Scheibner et al., *supra* note 3 at 27.

39
Personal Data Protection Commission, *supra* note 19 at 13.

40
Christine M. O’Keefe et al., De-Identification Decision-Making Framework 68 (2017), https://www.oaic.gov.au/privacy/guidance-and-advice/de-identification-decision-making-framework/.

41
*Supra* note 12 at Sections 13–14.

42
Personal Data Protection Commission, *supra* note 19 at 7.

43
*Supra* note 12 at 17.

44
*Id.* at Second Schedule, Part 2, Division 3.

45
*Id.* at Second Schedule, Part 3, Division 2.

46
Advisory Guidelines on Key Concepts in the PDPA, *supra* note 17 at 74–75.

47
*Supra* note 13 at Section 6.

48
*Id.* at Section 13(1)(b).

49
*Id.* at Fifth Schedule, paragraphs (a)–(b).

50
*Id.* at Fifth Schedule, paragraphs 3(c)–(d); Markus K. Labude, *A New Framework for Self-Regulation Legislation and Case Notes*, 2016 Sing. J. Legal Stud. 194, 205 (2016).

51
*Supra* note 13 at Fifth Schedule, paragraph 3(e).

52
Applicability of Consent Obligation to a Data Collaboration Agreement (2019), https://www.pdpc.gov.sg/-/media/Files/PDPC/PDF-Files/Practical-Guidance-Provided-by-PDPC/Guidance-to-organisations-involved-in-a-data-collaboration-agreement.pdf.

53
*Id.*

54
Practical Guidance to Queries by Medical Research Institution (2016), https://www.pdpc.gov.sg/-/media/Files/PDPC/PDF-Files/Practical-Guidance-Provided-by-PDPC/2016-07-medical-research-institution-pg.pdf.

55
*Supra* note 12 at Section 26.

56
Personal Data Protection Commission, *supra* note 19 at 23.

57 Personal Data Protection Regulations 2021, regulation 10(1).

58
*Id.* at Reg. 10(2)(a)–(b).

59
*Supra* note 12 at Schedule 1, Part 1, paragraph 1.; *supra* note 45 at regulation 10(2)(c).

60 Labude, *supra* note 38 at 205.

61
*Personal Data Protection Act 2012* (No. 26 of 2012), *supra* note 6 at Schedule 1, Part 2, paragraph 1; *Personal Data Protection Regulations 2021*, *supra* note 43 at regulation 10(2)(d)–(e)d.

62
*Supra* note 45 at regulation 9.

63
*Spize Concepts Pte Ltd* [2019] SGPDPC 22 25.

64
*Id.* at 26.

65
*Belden Singapore Privacy Limited* [2021] SGPDPC 13 17(a).

66
*Id.* at 24(a)–25.

67
*Toll Logistics (Asia) Limited and Others* [2022] SGPDPC 4.

68 Hui Yun Chan, Hui Jin Toh & Tamra Lysaght, *Cross-Jurisdictional Data Transfer in Health Research: Stakeholder Perceptions on the Role of Law*, ABR, 13–14 (2024), https://doi.org/10.1007/s41649-024-00283-8.

69
*Supra* note 45 at regulation 10(2)(a); Advisory Guidelines on Key Concepts in the PDPA, *supra* note 17 at 19.7.

70 Edward S. Dove, *The EU General Data Protection Regulation: Implications for International Scientific Research in the Digital Era*, 46 J Law Med Ethics 1013, 1026–1027 (2018).

71
Applicability of PDPA’s Consent Obligation in Relation to Transfer of Personal Data to Zuellig Pharma’s Trusted Execution Environment 1.1 (2023), https://www.pdpc.gov.sg/guidelines-and-consultation/2023/12/applicability-of-pdpas-consent-obligation-in-relation-to-transfer-of-personal-data-to-zuellig-pharmas-trusted-execution-environment.

72 Datenschutzgesetz *[Federal Act on Data Protection] 2023 SR 235.1*, Article 5(c).

73 Massimo Marelli, *The Law and Practice of International Organizations’ Interactions with Personal Data Protection Domestic Regulation: At the Crossroads between the International and Domestic Legal Orders*, 50 Comput. Law Secur. Rev. 105849, 14 (2023).

74 Humanforschungsgesetz *[Federal Human Research Act] 2014 SR 810.30*, Article 2.

75
*Supra* note 60 at Article 5(a).

76
*Regulation (EU) 2016/679 of the European Parliament and of the Council of 27 April 2016 on the Protection of Natural Persons with Regard to the Processing of Personal Data and on the Free Movement of Such Data, and Repealing Directive 95/46/EC (General Data Protection Regulation)*, Article 4(1).

77
Article 29 Data Protection Working Party, Opinion 04/2007 on the Concept of Personal Data (2007); Pablo Diaz, Data Protection: Legal Considerations for Research in Switzerland 4–5 (2022), https://iris.unil.ch/handle/iris/254606.

78
*Supra* note 62 at Article 3(f).

79
*Supra* note 60 at Article 6.4.

80 A Guide to Technical and Organisational Measures (2015), https://www.edoeb.admin.ch/dam/edoeb/de/Dokumente/aDSG/guideTOM_de.pdf.download.pdf/guideTOM_de.pdf.

81 Andrea Martani et al., *Data Protection and Biomedical Research in Switzerland: Setting the Record Straight*, 150 Swiss Med Wkly, 14 (2020), https://digitalcollection.zhaw.ch/handle/11475/21414.

82
*Supra* note 62 at Article 3(i).

83
Guidance Note: Guidance on Anonymisation and Pseudonymisation 5 (2019), https://www.dataprotection.ie/sites/default/files/uploads/2022-04/Anonymisation%20and%20Pseudonymisation%20-%20latest%20April%202022.pdf; Michèle Finck & Frank Pallas, *They Who Must Not Be Identified—Distinguishing Personal from Non-Personal Data under the GDPR*, 10 International Data Privacy Law 11, 16, 18–19 (2020).

84
*Research and Data Protection*, Federal Data Protection and Information Commissioner (FDPIC) (Jul. 23, 2024), https://www.edoeb.admin.ch/edoeb/en/home/datenschutz/forschung_statistik/forschung.html;  *Human Research*, Federal Data Protection and Information Commissioner (FDPIC) (Jul. 23, 2024), https://www.edoeb.admin.ch/edoeb/en/home/datenschutz/forschung_statistik/humanforschung.html.

85 Kelly E. Ormond et al., *What Are the Bottlenecks to Health Data Sharing in Switzerland? An Interview Study*, 154 Swiss Med Wkly 3538, 3–5 (2024).

86
*Supra* note 60 at Article 19.4.

87
*Id.* at Article 31(e).

88
*Id.* at Article 39.1.

89
*Supra* note 62 at Article 7.1.

90
*Id.* at 32.

91
*Id.* at 33.

92 Scheibner et al., *supra* note 3 at 7–8.

93
*Supra* note 62 at 34(a).

94
*Id.* at 34(b)–(c).

95 Stuart McLennan et al., *The Inconsistent Ethical Oversight of Healthcare Quality Data in Switzerland*, 148 Swiss Med Wkly, 2 (2018), https://smw.ch/article/doi/smw.2018.14637.

96 Scheibner et al., *supra* note 3 at 10; Flora Colledge, Sophie De Massougnes & Bernice Elger, *Consent Requirements for Research with Human Tissue: Swiss Ethics Committee Members Disagree*, 19 BMC Med. Ethics. 93, 4–6 (2018).

97
*Supra* note 60 at Article 5(e).

98 Datenschutzverodnung *[Federal Ordinance on Data Protection] 2023 SR 235.11*, Annex 1.

99
*Supra* note 60 at 17.1(a).

100
Guide to Checking the Direct or Indirect Data Transfers to Foreign Countries (Art. 6 Para. 2 Letter a FADP) (2021); The Transfer of Personal Data to a Country with an Inadequate Level of Data Protection Based on Recognised Standard Contractual Clauses and Model Contracts. (2021).

101
Guide to Checking the Direct or Indirect Data Transfers to Foreign Countries (Art. 6 para. 2 letter a FADP), *supra* note 88 at 4.

102
*Id.* at 5.

103
*Id.* at 6.

104 Benjamin Eze & Liam Peyton, *Systematic Literature Review on the Anonymization of High Dimensional Streaming Datasets for Health Data Sharing*, 63 Procedia Computer Science 348, 353 (2015).

105 Charu C. Aggarwal, *On K-Anonymity and the Curse of Dimensionality*, *in*  Proceedings of the 31st International Conference on Very Large Data Bases 901, 901–2 (2005).

106 Khaled El Emam et al., *The Re-Identification Risk of Canadians from Longitudinal Demographics*, 11 BMC Med. Inform. Decis. Mak. 46, 10 (2011).

107 Scheibner et al., *supra* note 3 at 28.

108 Chan et al., *supra* note 15 at 123.

109 Vincent Chow, *Businesses Navigating Data Transfer Uncertainties by Balancing Compliance Necessity, Business Costs*, China Law & Practice (2020).

110 Thi Tuyet Dung Thai, Van Duong Nguyen & Van Lam Nguyen, *A Comparative Study of Cross-Border Data Transfer Regulations in Vietnam and Singapore: Ensuring the Protection of Privacy*, 26 Asia-Pacific Journal on Human Rights and the Law 81, 97 (2025).

111 Sylvain Metille, *Swiss Information Privacy Law and the Transborder Flow of Personal Data*, 8 J. Int’t Com. L. & Tech. 71, 76 (2013).

112 Moira Paterson & Maeve McDonagh, *Data Protection in an Era of Big Data: The Challenges Posed by Big Personal Data*, 44 Monash U. L. Rev. 1, 11 (2018); Scheibner et al., *supra* note 3 at 12.

113 Mark Phillips, *International Data-Sharing Norms: From the OECD to the General Data Protection Regulation (GDPR)*, 137 Hum Genet 575, 578 (2018).

114 Emanuela Podda, *Shedding Light on the Legal Approach to Aggregate Data under the GDPR & the FFDR*, 3 (2021), https://cris.unibo.it/handle/11585/847902.

115 James Scheibner et al., *Revolutionizing Medical Data Sharing Using Advanced Privacy-Enhancing Technologies: Technical, Legal, and Ethical Synthesis*, 23 J. Med. Internet Res. e25120, 4 (2021).

116 Christoph Kurz, *Understanding Differential Privacy*, 18 Significance 24, 26 (2021).

117 Aloni Cohen & Kobbi Nissim, *Towards Formalizing the GDPR’s Notion of Singling out*, 117 Proc. Natl. Acad. Sci. U.S.A. 8344, 8346 (2020).

118 Georgios A. Kaissis et al., *Secure, Privacy-Preserving and Federated Machine Learning in Medical Imaging*, 2 Nat. Mach. Intell. 305, 306–309 (2020).

119 Dara Hallinan et al., *International Transfers of Personal Data for Health Research Following Schrems II: A Problem in Need of a Solution*, 29 Eur J Hum Genet 1502, 1505 (2021).

120 Bjørn Aslak Juliussen et al., *The Third Country Problem under the GDPR: Enhancing Protection of Data Transfers with Technology*, 13 International Data Privacy Law 225, 243 (2023).

121 Donrich Thaldar et al., *Communicating Clearly about Data Sharing in Genomics*, 19 Hum Genomics 80, 3 (2025).

122
*Id.* at 4.

123 Myriam Lingg & Verena Lütschg, *Health System Stakeholders’ Perspective on the Role of Mobile Health and Its Adoption in the Swiss Health System: Qualitative Study*, 8 JMIR mHealth and uHealth e17315, 2 (2020).

124 Heidi L. Rehm et al., *GA4GH: International Policies and Standards for Data Sharing across Genomic Research and Healthcare*, 1 Cell Genom, 12 (2021), https://www.cell.com/cell-genomics/abstract/S2666-979X(21)00036-7; Thaldar et al., *supra* note 109 at 4.

